# Supplementary material for: Ligand requirements for immunoreceptor triggering
Source: Commun Biol. 2024 Sep 13;7:1138. doi: 10.1038/s42003-024-06817-y (PMC11399299; doi:10.1038/s42003-024-06817-y)
Supplement: Supplementary file 2 — Supplementary Information [file 42003_2024_6817_MOESM2_ESM.pdf]

Supplementary Figures

Barton et al (2024) Ligand requirements for immunoreceptor triggering **Communications Biology**

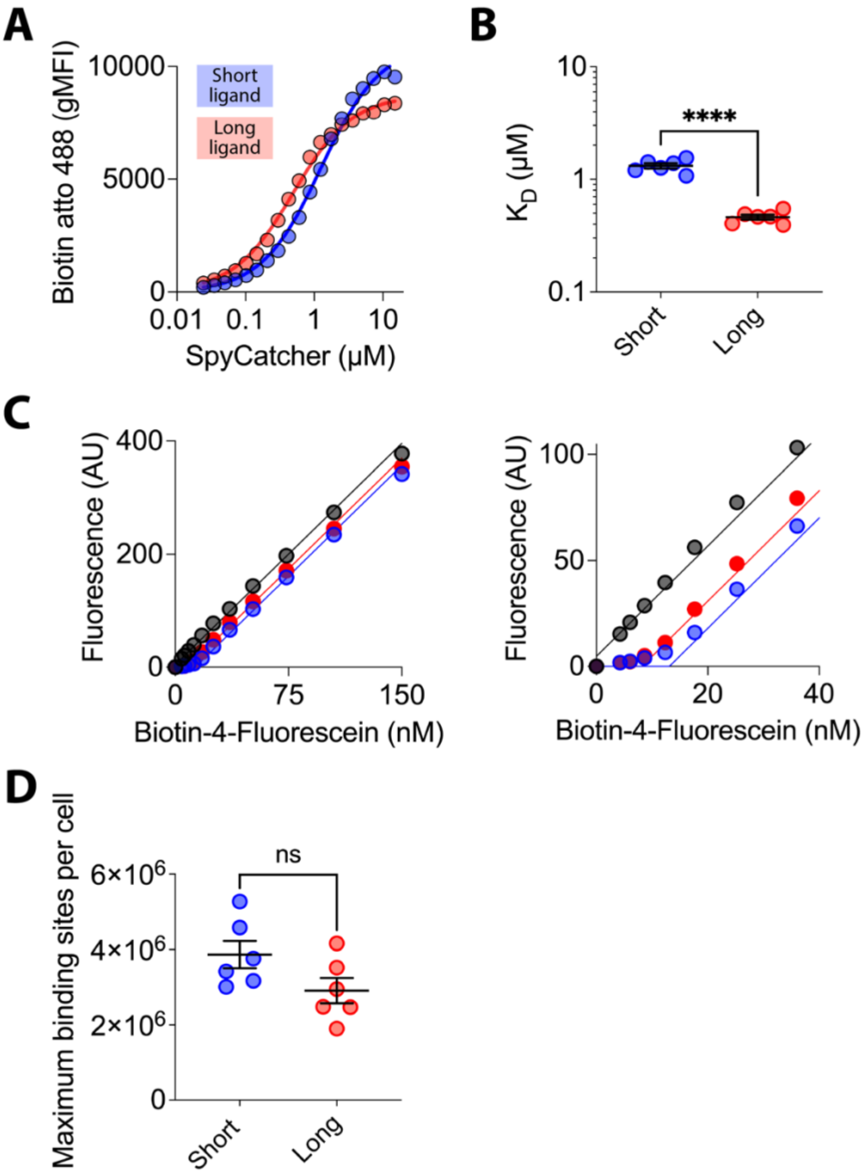

sFigure 1. Measuring the K_D and maximum number of binding sites on CHO cell expressing short and long CD80 ligand anchors. (A) Short (blue) or long (red) CD80 ligand CHO cells were incubated with the indicated concentration of monovalent StrepTactin SpyCatcher before labelling with biotin Atto 488 followed by flow cytometry. A one site simple binding model was fitted to the data (lines) to determine the K_D. (B) The mean and SD of K_D values from six independent experiments were compared using a t test. (C) Short or long CD80 ligand CHO cells or control (empty, grey) CHO cells were incubated with a saturating concentration (15 μM) of monovalent StrepTactin SpyCatcher before mixing with the indicated concentration of biotin-4-fluorescein and the unquenched fluorescence in the media measured after binding. (D) The mean and SD of the maximum number of binding sites per cell from six independent experiments were compared using a t test.

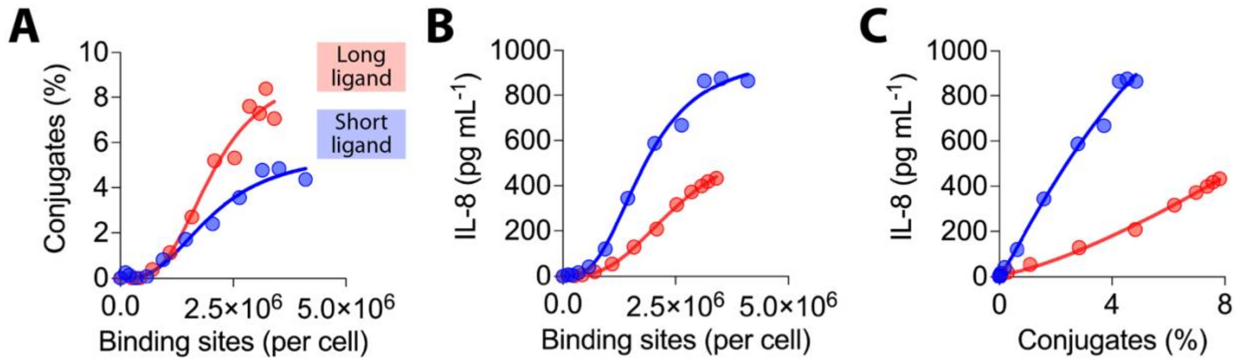

sFigure 2. Testing the effect of increasing ligand length on NTR activation. Repeat of experiment Figure 1 with both conjugation and stimulation assays performed on the same day with the same cells split. THP-1 cells expressing SIRPβ1 were incubated with CHO cells expressing the indicated numbers of short (blue) or long (red) generic ligand binding sites and conjugate formation (A) and IL-8 release (B) measured. Ligand binding sites were determined as described in the Materials and Methods using parameters determined in sFigure 1. The IL-8 release versus conjugation level is plotted in (C).

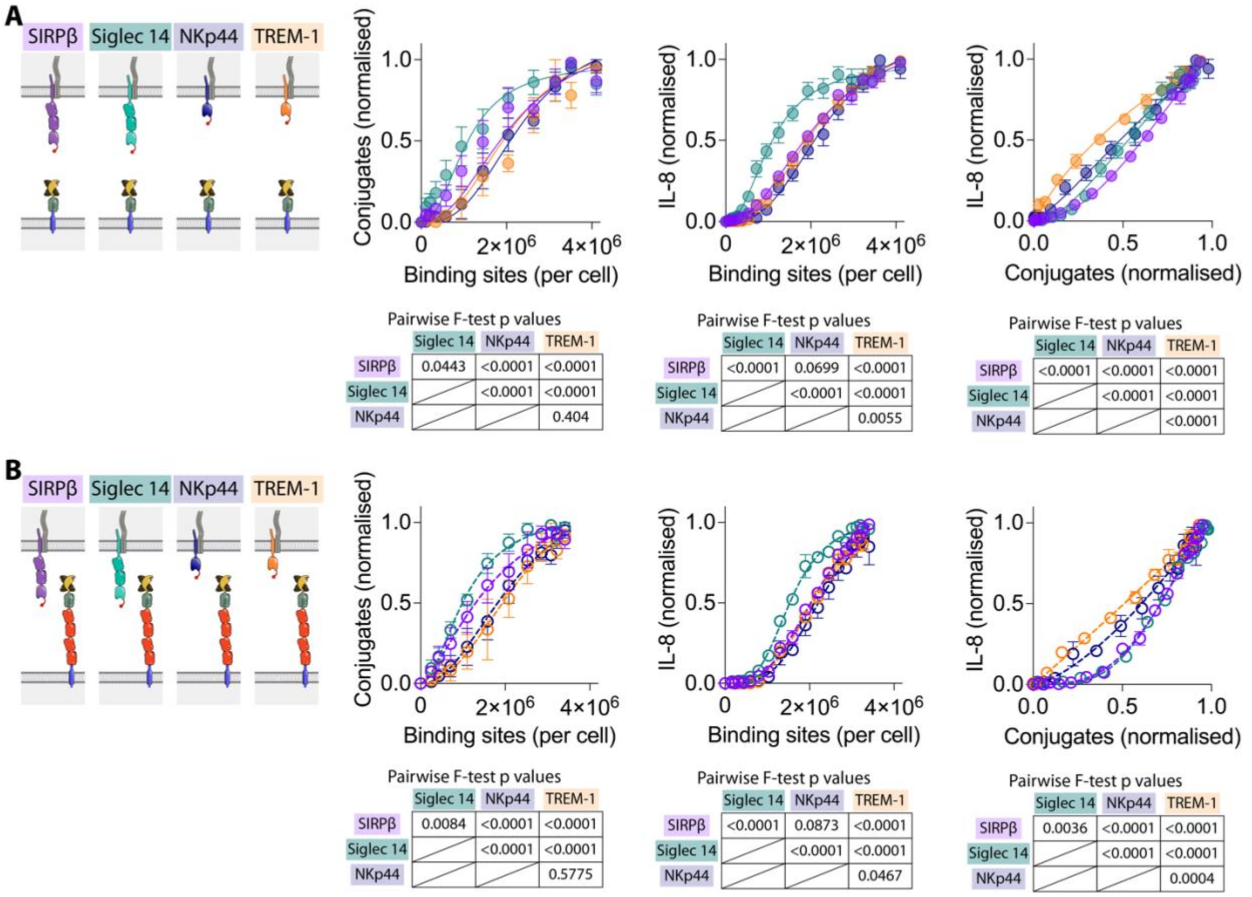

sFigure 3. The effect of NTR length on activation. The data from Figure 2 has been reanalysed to enable comparison between NTRs. THP-1 cells expressing the indicated NTR with N-terminal StrepTagII peptides were incubated with CHO cells expressing the indicated numbers of short (A) or long (B) generic ligand binding sites and conjugate formation (left panel) and IL-8 release (middle panel) measured. The IL-8 release versus conjugation level is plotted in the right panels. The data were fitted as described in the Materials and Methods and an F test was used to test the significance of differences between the fits collectively (panels) and pairwise (corresponding Tables).

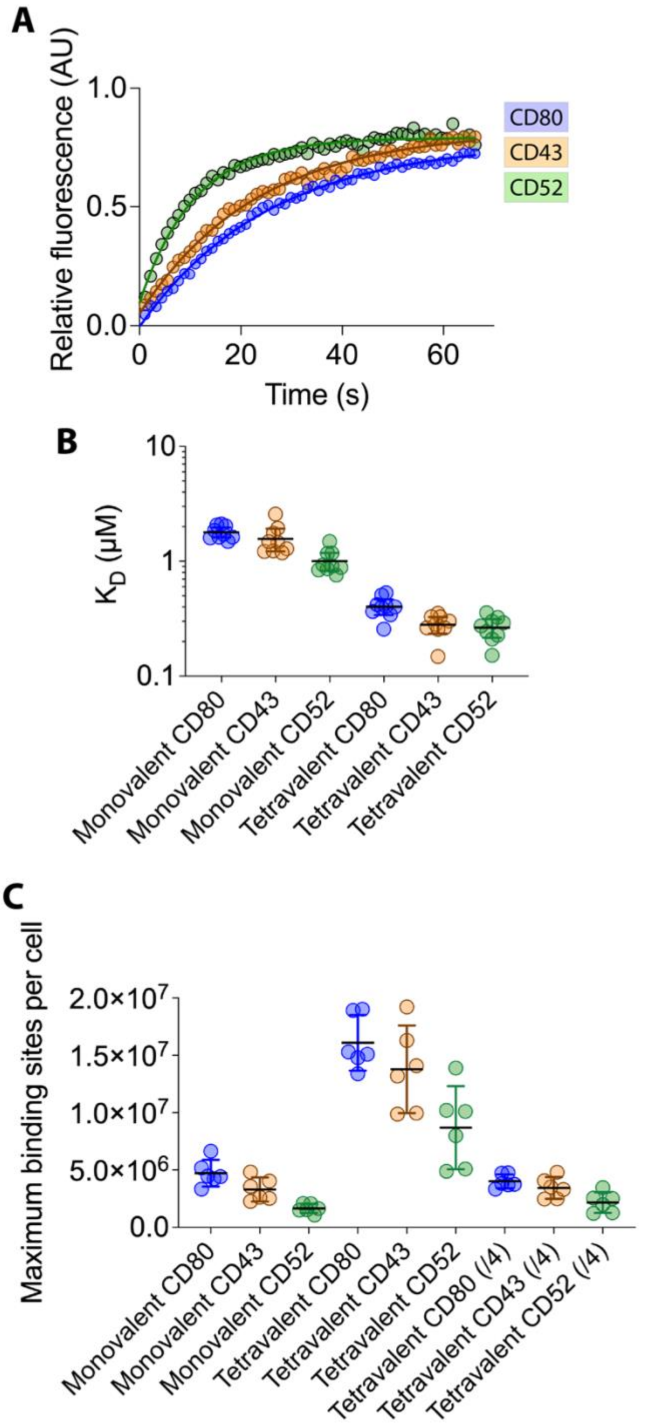

sFigure 4. Measuring mobility, K_D and the maximum generic binding sites on CHO cell expressing different ligand anchors. (A) CHO cells expression with the indicated generic ligand anchor were labelled with SpyCatcher-GFP and fluorescent recovery after 20 s photobleaching (FRAP) was performed. The recovery half-times, which were used to calculate diffusion coefficients, were determined from fits (lines) to the data (circles). (B) Mean and SD of K_D values determined as in sFigure 1 from nine independent experiments (C) Mean and SD of maximum number of binding sites determined as in sFigures 1 from six independent experiments. To enable comparison to monovalent sites the tetravalent values divided by four, labelled (/4), were also plotted.

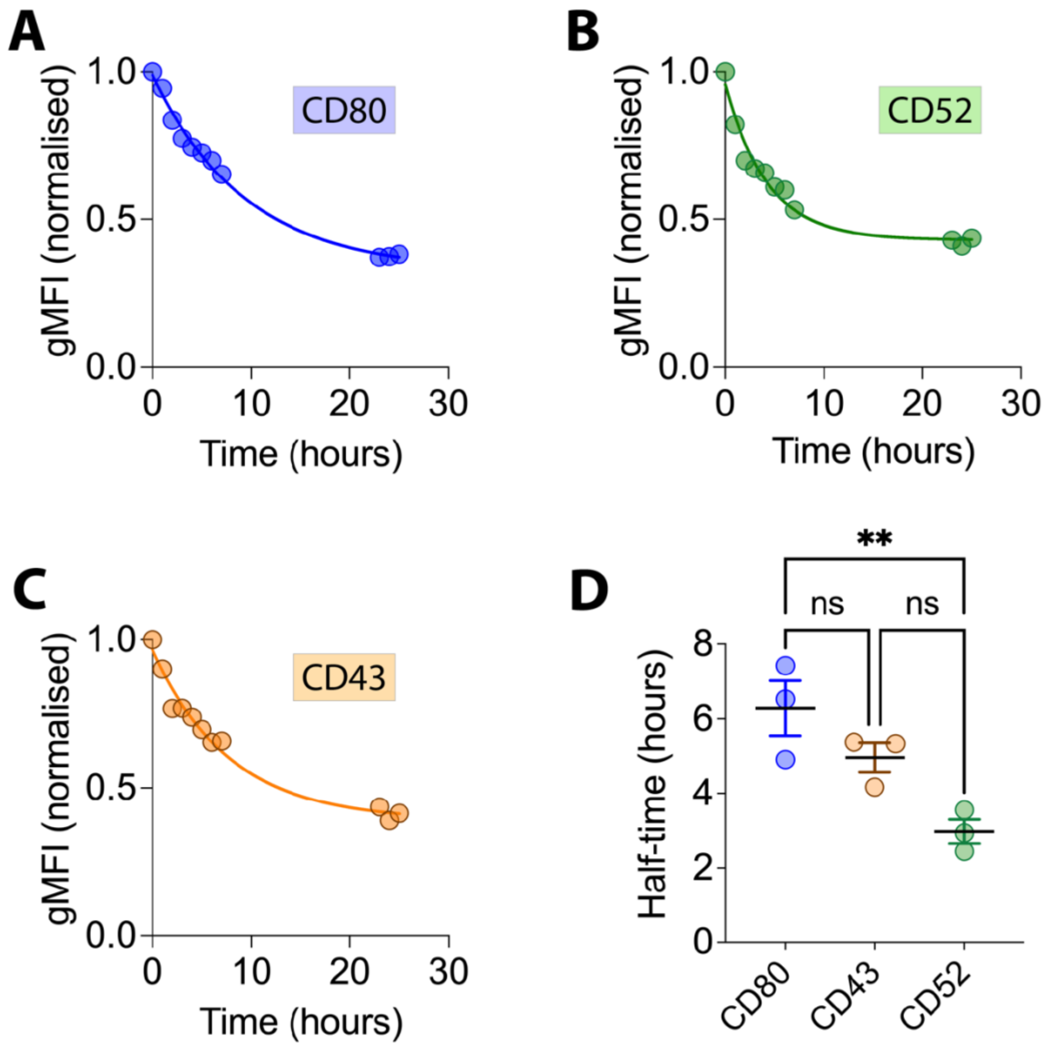

sFigure 5. Turnover of ligand anchor at the cell surface. (A-C) Monovalent StrepTactin SpyCatcher was coupled to CHO cells expressing the indicated ligand anchor and cells incubated at 37 C for the indicated times. Cells were stained with biotin ATTO 488 and analysed by flow cytometry to assess the level of the cell surface StrepTactin. The graphs show the relative level of ligand expression with the maximum normalised to time zero. The data is a representative of three biological replicates and was fit with an exponential one phase decay model. (D) The mean and SD of the half-time for loss from the cell surface for three biological replicates were compared by ANOVA.

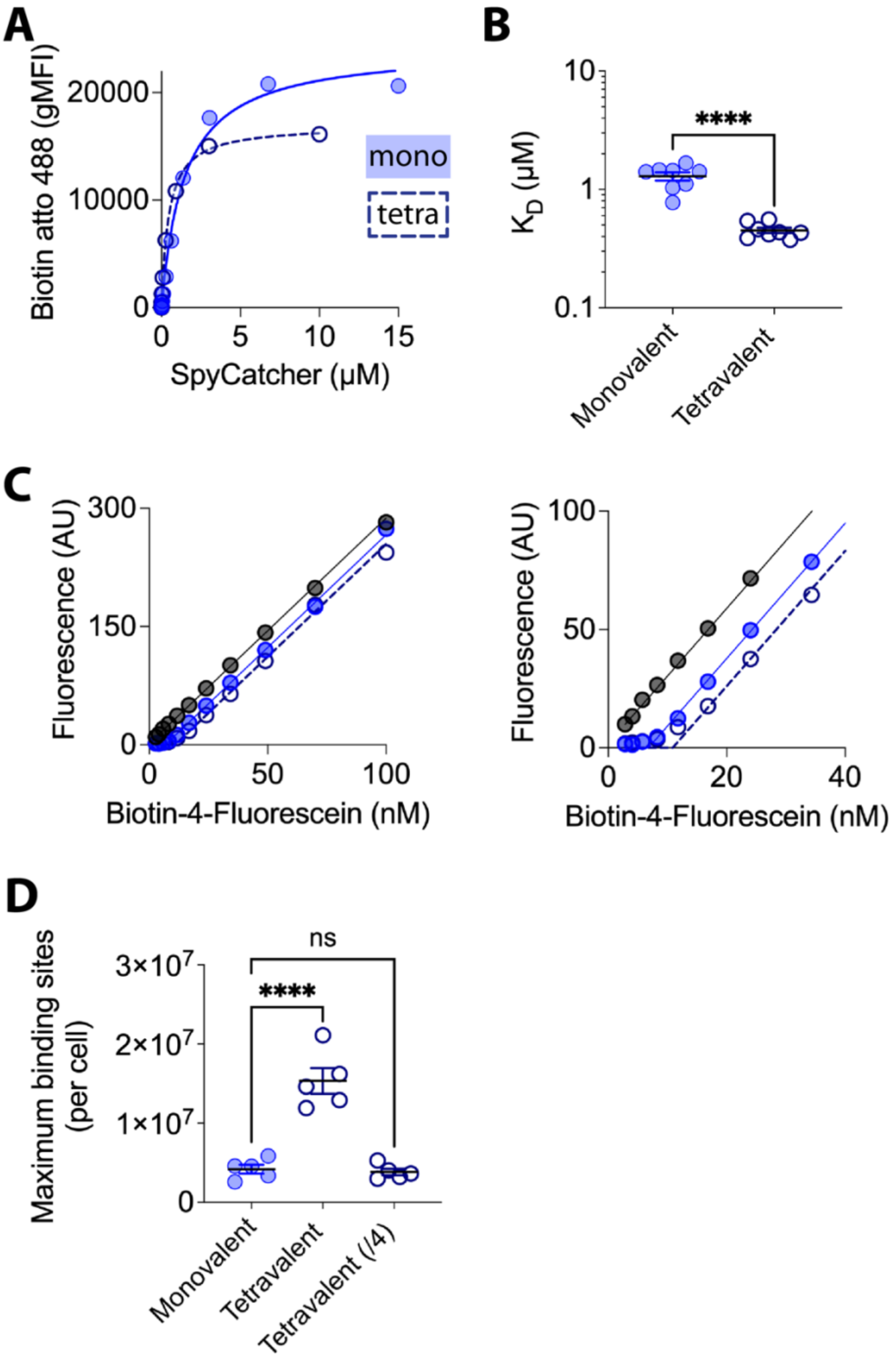

sFigure 6. Measuring the K_D and maximum number of binding sites on CHO cell presenting monovalent or tetravalent StrepTactin SpyCatcher. (A) Short CD80 ligand CHO cells were incubated with the indicated concentration of monovalent (closed circles) or tetravalent (open circles) StrepTactin SpyCatcher before labelling with biotin Atto 488 followed by flow cytometry. A one site simple binding model was fitted to the data (lines) to determine the K_D. (B) The mean and SD of K_D values from eight independent experiments were compared using a t test. (C) Short CD80 ligand CHO cells or control (empty, grey) CHO cells were incubated with a saturating concentration monovalent (10 µM) or tetravalent (10 µM) StrepTactin SpyCatcher before mixing with the indicated concentration of biotin-4-fluorescein and the unquenched florescence in the media measured after binding. (D) The mean and SD of the maximum number of binding sites per cell from five independent experiments were compared by ANOVA. To enable comparison to monovalent sites the tetravalent values divided by four were also plotted.

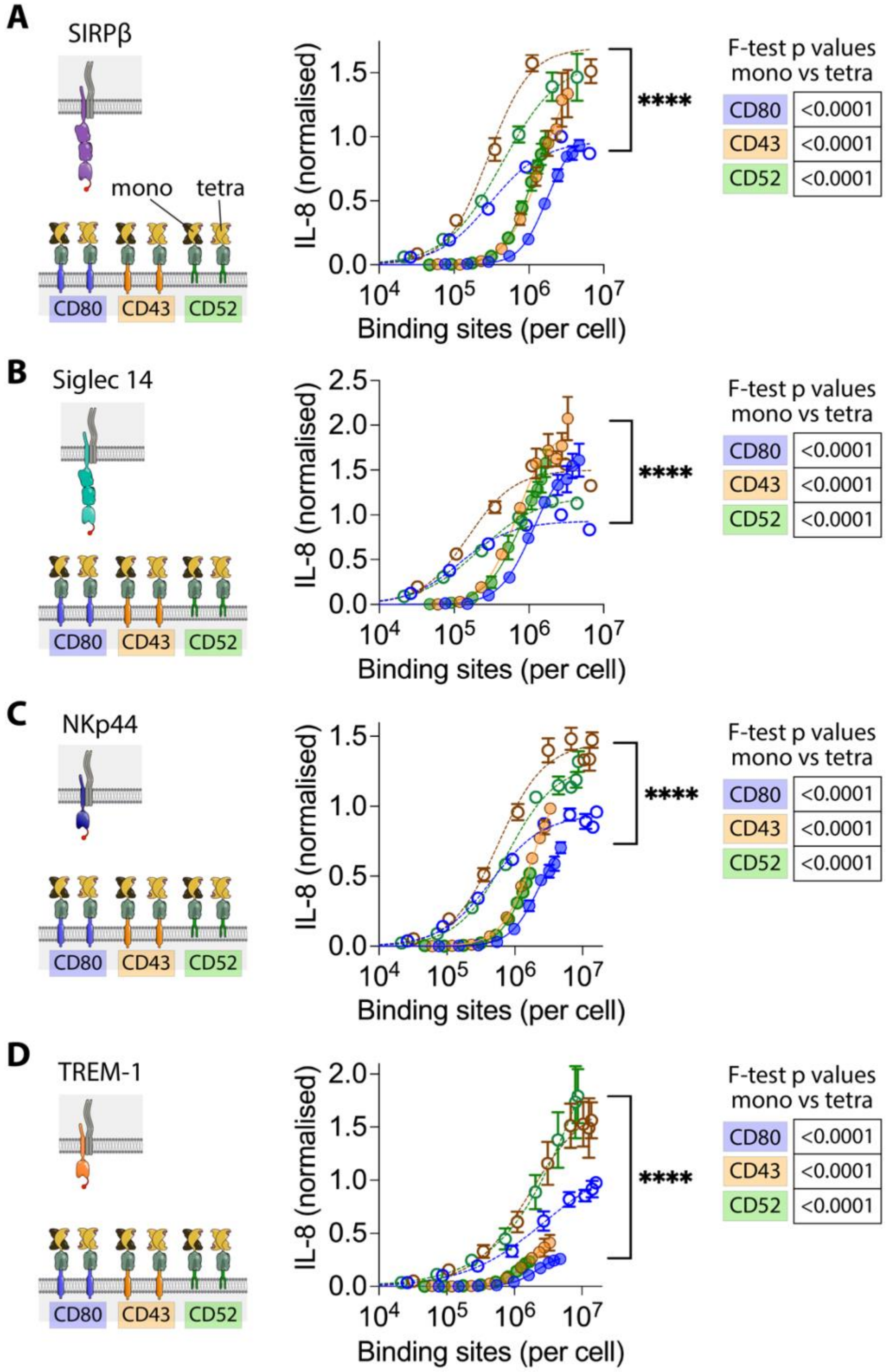

sFigure 7. Effect of anchor on NTR stimulation by multivalent ligand. THP-1 cells expressing (A) SIRPβ1, (B) Siglec 14, (C) NKp44 or (D) TREM-1 with an N-terminal StrepTagII peptide were incubated with CHO cells expressing the indicated number of monovalent (closed circles) or multivalent (open circles) ligand binding sites with the indicated ligand anchors and IL-8 release measured. Ligand binding sites were determined as described in the Materials and Methods using parameters determined in sFigure 4. The data from three biological replicates are plotted with the data normalised to the level of stimulation achieved with the CD80 ligand coupled with tetravalent ligand within each replicate. These data were fitted as described in the Material and Methods and an F test was used to test the significance of differences between the fits collectively and pairwise (Tables).

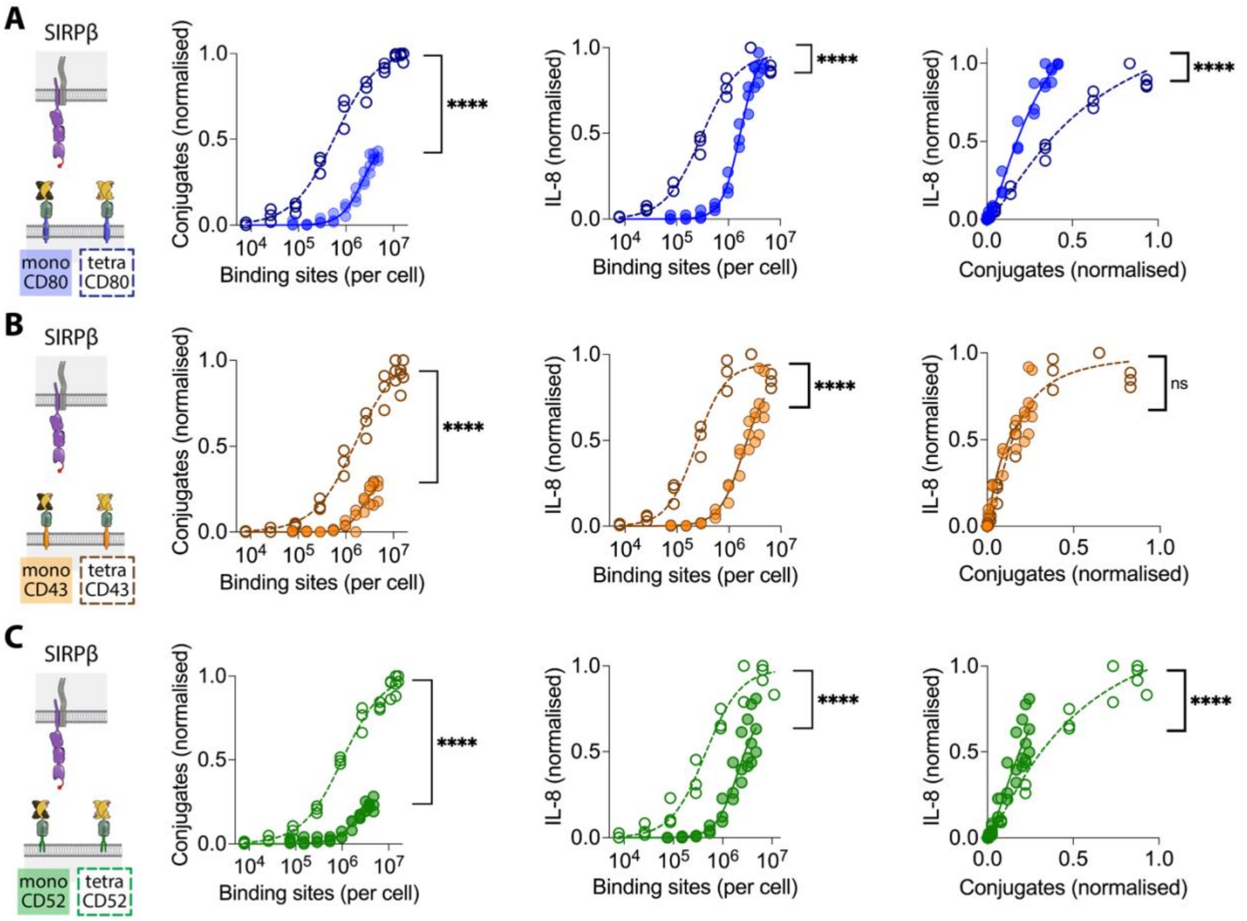

sFigure 8. Effect of anchor on SIRPβ1 stimulation by multivalent ligand. THP-1 cells expressing SIRPβ1 with an N-terminal StrepTagII peptide were incubated with CHO cells expressing the indicated number of ligand binding sites presented on monovalent (closed circles) or multivalent (open circles) ligand and conjugate formation (left panel) and IL-8 release (middle panel) were measured. Ligand binding sites were determined as described in the Materials and Methods using parameters determined in sFigure 4. The IL-8 release versus conjugation level is plotted in the right panel. The data from three biological replicates are plotted with the data normalised to the level of conjugation or stimulation achieved with the tetravalent ligand within each replicate. These data were fitted as described in the Material and Methods and an F test was used to test the significance of differences between the fits.

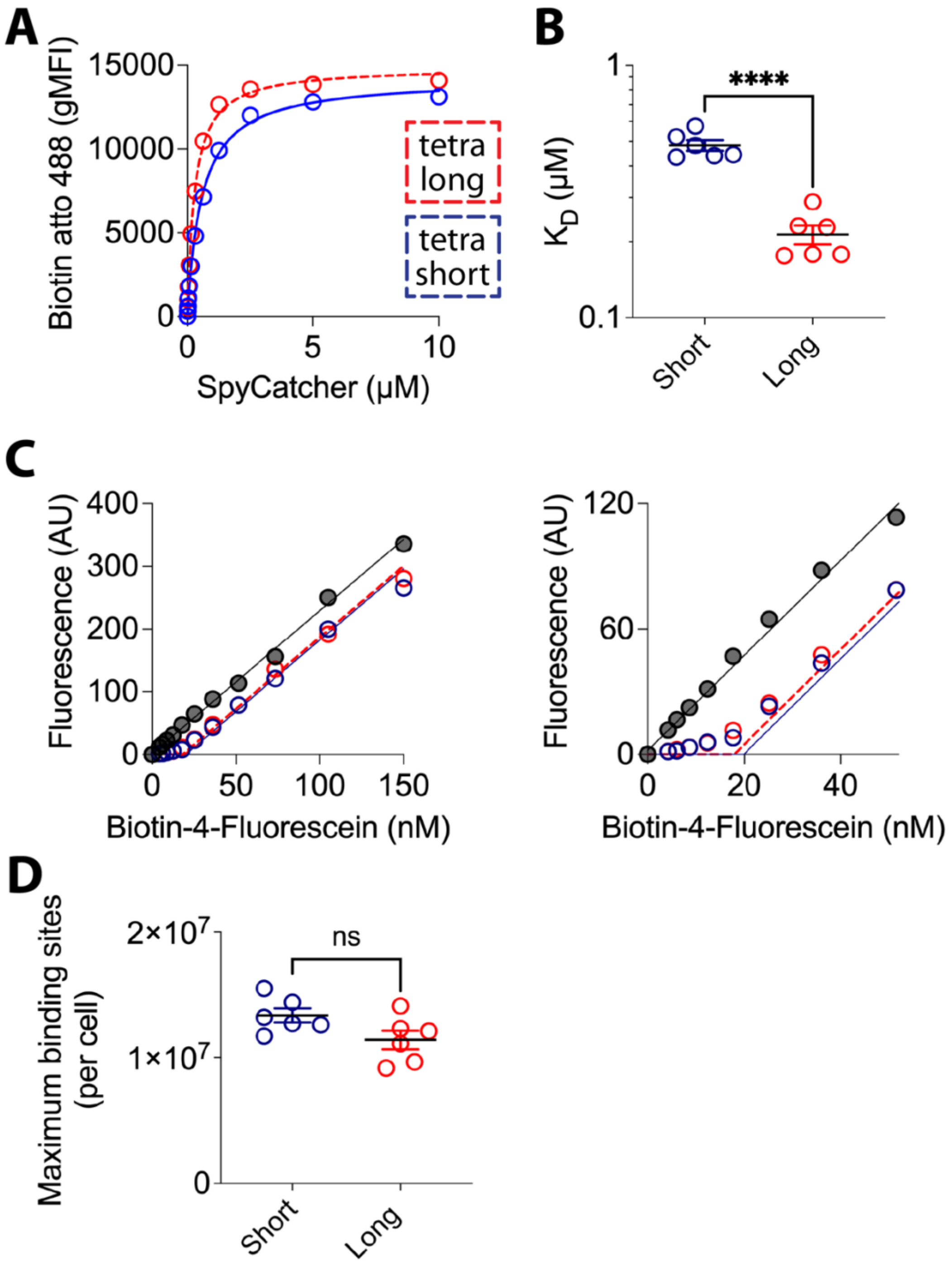

sFigure 9. Measuring the K_D and maximum number of binding sites on CHO cell presenting tetravalent StrepTactin SpyCatcher coupled to short versus long ligand anchors. (A) Short (blue) or long (red) CD80 ligand CHO cells were incubated with the indicated concentration of tetravalent StrepTactin SpyCatcher before labelling with biotin Atto 488 followed by flow cytometry. A one site simple binding model was fitted to the data (lines) to determine the K_D. (B) The mean and SD of K_D values from six independent experiments were compared using a t test. (C) Short or long CD80 ligand CHO cells or control (empty, grey) CHO cells were incubated with a saturating concentration (10 μM) of tetravalent StrepTactin SpyCatcher before mixing with the indicated concentration of biotin-4-fluorescein and the unquenched florescence in the media measured after binding. (D) The mean and SD of the maximum number of binding sites per cell from six independent experiments were compared using a t test.
