# Supplementary material for: Ligand requirements for immunoreceptor triggering
Source: Commun Biol. 2024 Sep 13;7:1138. doi: 10.1038/s42003-024-06817-y (PMC11399299; doi:10.1038/s42003-024-06817-y)
Supplement: Supplementary file 3 — Description of Additional Supplementary File [file 42003_2024_6817_MOESM3_ESM.pdf]

Description of Additional Supplementary Files

File: Supplementary Data
Description: Source data for the all the Figures in the paper.
